# Supplementary material for: A Systematic Scoping Review of Indigenous People’s Experience of Healing and Recovery from Child Sexual Abuse: A Protocol
Source: Int J Environ Res Public Health. 2024 Mar 7;21(3):311. doi: 10.3390/ijerph21030311 (PMC10970151; doi:10.3390/ijerph21030311)
Supplement: Supplementary file 1 [file ijerph-21-00311-s001.zip › ijerph-2810683-supplementary.pdf]

Category	Inclusion Criteria	Exclusion Criteria	Justification
**i.** Relevance to review's purpose	To be included in the review the publication/study must clearly outline in title, and/or abstract, and/or full-text stages to:<br>**1.** have a primary and explicit focus, **or**<br>**2.** be largely inclusive of an explicit focus on:<br>***a.*** the healing and recovery experience of Indigenous survivors of CSA, **or**<br>***b.*** the cultural components of healing and recovery for Indigenous survivors of CSA<br>***c.*** the factors related to the barriers to and enablers of disclosure for Indigenous survivors of CSA **or**<br>***d.*** the factors related to the barriers to and enablers of access and engagement with services for Indigenous survivors of CSA.	Any publication/study that is determined by the reviewers to not clearly outline in title, abstract and/or full-text stages to:<br>**1.** not have a primary focus, **or**<br>**2.** not be inclusive of a focus on:<br>***a.*** the listed inclusion criteria, **or**<br>**3.** to primarily focus on an issue that is outside the scope of this review is to be excluded.	While this review is broadly inclusive, all literature must pertain to the primary research topic, which is the collation, extraction and synthesis of current documented academic and industry literary evidence regarding the healing and recovery of Indigenous survivors of CSA, potential impacting factors, and associated barriers to and enablers of healing. Any literature that is considered to be outside this scope is to be excluded. For example, literature in which childhood trauma is discussed, but does not clearly indicate the specific inclusion of childhood sexual abuse will not be considered to meet the scope of this review.
Publication Characteristics			
**ii.** Publication date	To be included in the review, the publication must have been published between 1990 and the current date of search (inclusive).	Any publication that was published prior to or during 1989 is to be excluded from the review.	Publications within the last 30+ years provide a wide breadth of literature. A wide breadth of literature allows for analysis of a good scope and account for systemic or factors that are impactful over long periods of time. No previous systematic literature reviews within this period (or prior).
**iii.** Publication type	To be included in the review the publication must be **one of** the following:<br>**1.** journal article<br>**2.** industry or governmental report<br>**3.** book<br>**4.** book chapter<br>**5.** archival material<br>**6.** personal journals and diaries<br>**7.** case notes<br>**8.** any other documents that are not listed as exclusion criteria.	Any publication that is determined by the reviewers to be **one of** the following is to be excluded:<br>**1.** abstracts<br>**2.** protocols<br>**3.** reviews<br>**4.** opinion pieces	Publications in the inclusion criteria provide a potential depth of content that is above that of the publications in the exclusion criteria.

Category	Inclusion Criteria	Exclusion Criteria	Justification
**iv.** Publication location	All publication locations are acceptable for inclusion.	No publication locations are excluded.	Publication location is not considered part of this review. Study location and population are considered more appropriate for the purpose of this review. Studies that are inclusive of the target population (and therefore within the scope) may be published in international journals, and as such, they cannot be excluded.
**v.** Scholarly publication/peer review	To be included in the review, the publication must be considered scholarly or appropriately representative of Indigenous knowledge by the reviewers such that:<br>**1.** journal articles must be:<br>***a.*** peer-reviewed, and<br>**2.** all publications must be identified by the reviewers as meeting ***all*** of the following:<br>***a.*** is written for researchers, scholars or professionals in the article's respective field<br>or<br>is written for the general public, but sufficiently represents the views and voices of Indigenous peoples<br>***b.*** is inclusive of technical, academic or detailed information on the topic<br>***c.*** is written in a style that is common to or similar to other technical or scholarly publications on the topic<br>**or**<br>is written from an Indigenous viewpoint, or with sufficient representation of Indigenous voices to be able to fairly represent an Indigenous viewpoint<br>***d.*** presents detailed information in a way that is considered to be sufficiently accurate or sufficiently identifies potential bias<br>***e.*** has sources always cited in a scholarly and accurate manner.	Any publication/study that meets the publication type criteria but is determined by the reviewers to not meet the inclusion criteria listed to be a scholarly publication are to be excluded such that:<br>**1.** journal articles are not:<br>***a.*** peer-reviewed, and<br>**2.** all publications are identified by the reviewers as not meeting ***all*** of the following:<br>***a.*** written for researchers, scholars or professionals in the article's respective field<br>or<br>written for the general public but sufficiently represents the view and voices of indigenous peoples.<br>***b.*** is inclusive of technical, academic or detailed information on the topic<br>***c.*** is written in a style that is common to or similar to other technical or scholarly publications on the topic<br>**or**<br>is written from an indigenous viewpoint or with sufficient representation of indigenous voices to be able to fairly represent an indigenous viewpoint.<br>***d.*** presents detailed information in a way that is considered to be sufficiently accurate or sufficiently identifies potential bias<br>***e.*** has sources always cited in a scholarly and accurate manner.	Publications that have been identified as peer-reviewed or scholarly (by having met the listed criteria) allows for more confidence in the accuracy and bias of the included publications. Publications that are inclusive or primarily from an Indigenous viewpoint or Indigenous voices allow for a better understanding of the direct experiences of Indigenous survivors and the impacts of culture. Excluded publications are considered by the reviewers to be less accurate, have greater risk of bias, or not sufficiently include the experiences of Indigenous perspectives.
Study Characteristics			

Category	Inclusion Criteria	Exclusion Criteria	Justification
**vi.** Data or content type	Studies that meet the criteria for inclusion use:<br>**1.** qualitative data<br>**2.** quantitative data, and<br>**3.** theoretical or literary analyses, when the data meet **all** of the following:<br>***a.*** used for original research<br>***b.*** to formulate original conclusions, ***and*** are<br>***c.*** not used for the purpose of summarisation or review<br>**4.** personal accounts, when it is clearly the personal account of an Indigenous person who has lived experience of CSA, or the healing and recovery of Indigenous peoples who have experienced CSA.	Studies that are to be excluded are:<br>**1.** studies that use their data for the purpose of unoriginal research, including summarisation or review<br>**2.** studies that use data that is not original, or<br>**3.** used in multiple publications, **unless** that data is:<br>***a.*** used for novel or unique conclusions from the other publications and therefore meets the inclusion criteria **3*a*** and **3*b***.<br>**4.** studies that do not include data that clearly relate to the research questions.	All data types are included, as this provides a better scope of the current state of the literature and allows for both a statistical understanding and a qualitative examination of Indigenous survivors' experiences of healing and recovery from CSA, related factors and barriers/enablers.<br>Exclusion criteria highlight the importance of original and up-to-date uses of data. Data that are used in multiple publications that do not provide novel or unique conclusions do not provide additional content for the purpose of this review.
**vii.** Study design/methodology	All study designs and/or methodologies are eligible for inclusion.	No study designs and/or methodologies are excluded.	To maintain inclusivity in this review, no study designs and/or methodologies will be excluded.

Category	Inclusion Criteria	Exclusion Criteria	Justification
**viii.** Study population (PCC)	To be included, the targeted study population must include:<br>**1.** Indigenous peoples from one of the six Indigenous groups, which include:<br>***a.:*** Australian Aboriginal and Torres Strait Islander peoples, or<br>***b.*** New Zealand Māori peoples<br>***c.*** Canada's First Nations peoples, Inuit and Métis peoples<br>***d.*** North America's Native American peoples<br>***e.*** Alaska's Native peoples<br>***f.*** Sámi peoples of the Sápmi region in Northern Europe.<br>**2.** Studies that include multiple populations (that may include populations in the exclusion criteria) may be included if:<br>***a.*** the study provides novel/original data or conclusions related to the review purpose, **and**<br>***b.*** the data/conclusions are based, in part, on/or directed at one or more of the included study populations listed in Inclusion **viii.1*a*–f**, **and**<br>***c.*** the demographics and subsequent findings are generalisable to the relevant population/s as per Inclusion **viii.2*b***. This may be by having a sufficient statistical representation that is accurately representative of the relevant population in relation to other included populations within the chosen study/document.	Studies that are to be excluded are:<br>**1.** those studies whose target population is wholly:<br>***a.*** non-Indigenous, or<br>***b.*** from an Indigenous group not listed in Inclusion **viii.1*a*–f**.<br>**2.** Studies that include multiple populations and include an included study population but **do not:**<br>***a.*** provide novel/original data, or<br>***b.*** draw conclusions relevant to the study purpose, or<br>***c.*** provide sufficiently generalisable or representative data of or findings to the relevant Indigenous group/s.	In order to ensure specificity of the review, it is important to target the PCC critically. While aspects of this review are kept broad to ensure a wide capture of literature, the target population is kept very narrow. This allows for more focused and specific conclusions that may best inform future research, policy and practice. Key excluded populations are:<br>**Non-Indigenous peoples**. The purpose of this review is to critically examine the literature to identify the key factors associated with healing for Indigenous survivors of CSA to inform future therapeutic practices, programs and policy that address healing from CSA. As such, literature that does not have a focus on Indigenous peoples does not support that purpose.<br>**Indigenous peoples that are not a part of the listed included groups**. While many unique Indigenous groups exist, the six listed groups often represent the most researched and have the most Indigenous-specific and -led research. The research team believe that by specifically targeting these groups, they can maintain specificity and relevance while not losing valuable research. Further, the listed groups show similar colonial experiences, modern political and social conditions, and similar barriers to and enablers of access to healthcare and mental health support.

Category	Inclusion Criteria	Exclusion Criteria	Justification
**ix.** Study concept (PCC)	To be included, the target study must have a primary focus on **one or more** of the following concepts in regard to the included populations:<br>**1.** the experience of healing and recovery of Indigenous survivors of CSA<br>**2.** any cultural components of healing and recovery for Indigenous survivors of CSA<br>**3.** the barriers to and enablers of disclosing the experience of CSA for Indigenous survivors<br>**4.** the barriers to and enablers of accessing and engaging with healing and recovery services for Indigenous survivors.	Studies are to be excluded if they do not include any of the inclusion criteria **and/or:**<br>**1.** the factor or concept occurs relates to survivors of CSA but does not clearly relate to one of the included Indigenous populations<br>**2.** the study does not include a primary focus on one of the included concepts; for example, the study should be able to demonstrate that one of the included concepts informed the purpose of the study, its design, methods, results and conclusions.	The use of a concept focus allows for the study to broadly identify the breadth of potential factors that have implications for the research, practice and provision of healing and recovery for Indigenous peoples. Because this is a large concept, to be included, any and all studies must be able to demonstrate their clear targeted focus on the concept of healing and recovery for Indigenous survivors of CSA.
**x.** Study context (PCC)	This review will broadly capture contexts in which Indigenous peoples experience healing from CSA. As such, no specific contexts will be excluded should inclusion criteria **viii** and **ix** be met. Some examples of potential contexts include, but are not limited to:<br>**1.** targeted/focused Indigenous CSA interventions (individual and group)<br>**2.** broader well-being and mental health interventions that specify healing from CSA as an outcome<br>**3.** community programs and evaluations<br>**4.** institutional programs and evaluations<br>**5.** governmental enquiries.	No contexts in which healing may occur will be excluded. As such, studies will only be excluded based on context. Rather, the relevance of the context will be determined according to how it informs inclusion and exclusion criteria **viii** and **xi**. Should a study's population or concept be difficult to determine, context may be considered in group discussion to inform exclusion, on the grounds that:<br>**1.** the context provides reasonable grounds to determine the study is not relevant to Indigenous peoples, i.e., the intervention occurs outside the geographical region where included Indigenous groups may normally seek healing.<br>**2.** the context provides reasonable grounds to determine that the study concept is not relevant to the study, i.e., the context relates to an intersectional issue such as an AOD rehabilitation facility, but does not clearly specify the relevance of CSA.	Healing can occur at any age, over any timeframe and within many settings. As such, there are few grounds to exclude based on contextual factors, unless those contextual factors can better inform the relevance of other PCC criteria.

KEY DEFINITIONS

Primary Focus: The document clearly and explicitly identifies in its title, abstract, or full text that its aims, hypotheses, procedures, target populations, concept, or outcomes have a majority focus on the screened criteria. For example, a project that details that its aim is to explore the stories of **Aboriginal and Torres Strait Islander women's experiences of disclosure of CSA** to doctors in hospital settings in Victoria, and its procedures included qualitative interviews that are analysed thematically to capture barriers to and enablers of disclosure, the impact of disbelief on service access, and the experience of racism, then this study would have a primary focus on Aboriginal and Torres Strait Islander Australian women, child sexual abuse, barriers and enablers to disclosure, and barriers and enablers to accessing and engaging with services.

Partial Focus: The document clearly and explicitly identifies in its title, abstract, or full text that its aims, hypotheses, procedures, target populations, concept, or outcomes have included some information regarding the screened criteria. For example, a project titled 'The impact of Interpersonal Therapy on Canadian Adolescents with a diagnosis of persistent depression who have experienced sexual assault' would be screened in the title stage as included due to its partial focus on the healing and recovery from sexual abuse in one of the identified geographical areas. Through exploration of its abstract, if it identified that it included First Nations Canadian, Metis, or Inuit participants, this would indicate a partial focus. Full-text screening would be appropriate based on this partial focus.

PUBLICATION INFORMATION

All publications are first to be reviewed for inclusion and exclusion based on publication information to ensure that only relevant publication types undergo screening.
Note criteria **ii**, **iii**, **iv** & **v**: If the publication meets inclusion criteria for criteria **iii. 2–4**, then it may be included without full review of criteria **v. 2. a–e.** These criteria will be considered if the article makes it to abstract/full-text screening. Reason for exclusion at this stage should be noted in EndNote column 'Reason for Exclusion' as 'Publication Characteristics'.

SCREENING

1. TITLE SCREENING
Titles are to be screened first and included only with the clear identification of criteria: **i**, **viii**, **ix**. Titles that seem ambiguous or appear to somewhat meet the criteria may be considered for discussion. Reason for exclusion at this stage should be noted in EndNote column 'Reason for Exclusion' by the specific criteria for exclusion as per the above table, e.g., i. Relevance to review purpose.

2. ABSTRACT SCREENING
Abstracts are to be screened with consideration of all criteria where possible. Any ambiguity or lack of clarity for any criteria can be resolved via team discussion. The primary reason for exclusion at this stage should be noted in EndNote column 'Reason for Exclusion' by the specific criteria for exclusion as per the above table, e.g., i. Relevance to review purpose. Any additional exclusion criteria may be noted in EndNote column 'Additional Notes' by their numeral (i–x).

3. FULL-TEXT SCREENING
Full texts are to be screened with consideration of all criteria where possible. Any ambiguity or lack of clarity for any criteria can be resolved via team discussion. The primary reason for exclusion at this stage should be noted in EndNote column 'Reason for Exclusion' by the specific criteria for exclusion as per the above table, e.g., i. Relevance to review purpose. Any additional exclusion criteria may be noted in the EndNote column 'Additional Notes' by their numeral (i–x).
